# Association of DRD4 uVNTR and TP53 codon 72 polymorphisms with schizophrenia: a case-control study

**DOI:** 10.1186/1471-2350-10-147

**Published:** 2009-12-29

**Authors:** For-Wey Lung, Bih-Ching Shu, Wei-Tsung Kao, C Nathan Chen, Yu-Chi Ku, Dong-Sheng Tzeng

**Affiliations:** 1 Department of Psychiatry, Kaohsiung Armed Forces General Hospital, Kaohsiung, Taiwan; 2 Department of Neurology, Kaohsiung Medical University, Kaohsiung, Taiwan; 3 Department of Psychiatry, National Defense Medical Centre, Taipei, Taiwan; 4 Calo Psychiatric Centre, Pingtung County, Taiwan; 5 Institute of Allied Health Sciences and Department of Nursing, National Cheng Kung University, Tainan, Taiwan; 6 Graduate School of Human Sexuality, Shu-Te University, Kaohsiung County, Taiwan; 7 CNS Marketing, Janssen-Cilag, Taipei, Taiwan; 8 Department of Psychiatry, National Cheng Kung University, Tainan, Taiwan; 9 Graduate Institute of Occupational Safety and Health, Kaohsiung Medical University, Kaohsiung, Taiwan; 10 Institute of Undersea and Hyperbaric Medicine, National Defense Medical Centre, Taipei, Taiwan; 11 Beitou Armed Forces Hospital, Taipei, Taiwan

## Abstract

**Background:**

The tumour supressor gene TP53 is thought to be involved in neural apoptosis. The polymorphism at codon 72 in TP53 and the long form variants of the upstream variable number of tandem repeats (uVNTR) polymorphism in the dopamine D4 receptor (DRD4) gene are reported to confer susceptibility to schizophrenia.

**Methods:**

We recruited 934 patients with schizophrenia and 433 healthy individuals, and genotyped the locus of the TP53 codon 72 and DRD4 uVNTR polymorphisms by combining the polymerase chain reaction-restriction fragment length polymorphism method (PCR-RFLP) with direct sequencing.

**Results:**

No significant differences were found in the frequency of the genotype of the TP53 codon72 polymorphism between patients with schizophrenia and their controls. However, the long form alleles (≥ 5 repeats) of the DRD4 uVNTR polymorphism were more frequent in patients with schizophrenia than in controls (p = 0.001). Hence, this class of alleles might be a risk factor for enhanced vulnerability to schizophrenia (odds ratio = 3.189, 95% confidence interval = 1.535-6.622). In the logistic regression analysis, the long form variants of the DRD4 polymorphism did predict schizophrenia after the contributions of the age and gender of the subjects were included (p = 0.036, OR = 2.319), but the CC and GG genotypes of the codon 72 polymorphism of TP53 did not.

**Conclusions:**

The long form variants of the uVNTR polymorphism in DRD4 were associated with schizophrenia, in a manner that was independent of the TP53 codon 72 polymorphism. In addition, given that the genetic effect of the TP53 codon 72 polymorphism on the risk of developing schizophrenia was very small, this polymorphism is unlikely to be associated with schizophrenia. The roles that other single nucleotide polymorphisms (SNPs) in the TP53 gene or in other apoptosis-related genes play in the synaptic dysfunction involved in the pathogenesis of schizophrenia should be investigated.

## Background

The results of neuropsychological and neuroimaging studies suggest that abnormal connections between various cortical and subcortical regions of the brain play an important role in the pathogenesis of schizophrenia [1,2]. During the last two decades, remarkable progress has been made in identifying changes in the brain that are related to the pathophysiology of schizophrenia. Although the aetiology remains unknown, several convergent findings suggest that disruption of the cortical synaptic circuitry is a central defect in schizophrenia; these include the progressive loss of cortical gray matter during the first episode of psychosis, reduced synaptic markers, reduced neuropil, and reductions in neurons that are specific to cortical layers [3]. The underlying mechanisms that lead to synaptic dysfunction in patients with schizophrenia remain unknown; however, dysregulation of neuronal apoptosis has been suggested to contribute to the pathophysiology of the disease [4,5]. The pro-apoptotic events that occur within the brains of patients with schizophrenia do not affect the number of cortical neurons in the prefrontal cortex [6,7], but do give rise to a reduction of neuropil that is accompanied by a high neuronal density [8].

Among the proteins that are related to apoptosis, the level of Bcl-2 is reduced by 25% in the middle temporal gyrus in patients with schizophrenia as compared with controls. A high Bax/Bcl-2 ratio is also detected in the neurons and glia of the temporal cortex of patients with schizophrenia [9], which suggests that pro-apoptotic stimuli might be more likely to lead to the uncontrolled release of cytochrome c in these cells than in normal cells. The excess cytochrome c is released into the cytosol where it initiates the caspase cascade [10,11]. The well-known tumour suppressor p53, which is encoded by the TP53 gene, has been proposed to be an upstream regulator of the intrinsic apoptotic pathway, which is mediated by Bax [12]. In general, in response to DNA damage, p53 triggers either growth arrest through the cell cycle regulator p21 and/or apoptosis via PUMA-Bax signalling [13]. Moreover, it has been shown that increased TP53 expression in the mouse embryonic brain results in neuronal damage [14]. It has also been observed that the TP53 gene acts to control the elimination of cells with genetic abnormalities; for example, p53-mediated neuronal apoptosis is induced in patients with schizophrenia [15,16]. Therefore, it was of interest to determine whether the TP53 gene confers vulnerability to schizophrenia. The TP53 codon 72 polymorphism, an arginine (Arg; CGC)/proline (Pro; CCC) substitution polymorphism at codon 72 of TP53, is speculated to affect the induction of apoptosis by p53. The two genetic variants have been reported to function differently: the Arg72 variant is considered to trigger apoptosis more efficiently than the Pro72 variant. Moreover, the Arg72 variant is localised to mitochondria and induces the release of excess cytochrome c into the cytosol [17]. This polymorphism is also reported to be associated with vulnerability to cancer: the Pro/Pro genotype is a risk factor associated with enhanced vulnerability to epithelial cancers, such as lung cancer (odds ratio (OR) = 2.98) [18], colorectal cancer (OR = 1.699) [19], transitional cell carcinoma [20], and cervical cancer [21]. However, it remains unclear whether this polymorphism is associated with schizophrenia.

The D4 dopamine receptor (DRD4) gene contains an upstream variable number of tandem repeats (uVNTR) polymorphism that comprises between two and 10 repeats. The polymorphism is located in exon 3 of the gene, which encodes the cytoplasmic portion of the receptor and is critical for its biological function [22,23]. According to experimental evidence from functional studies, the two-repeat and four-repeat variants are associated with a greater reduction in cyclic adenyl monophosphate (cAMP) levels upon binding to dopamine than the seven-repeat variant [9,24]. This polymorphism has been linked to variations in dopaminergic signalling and confers susceptibility to schizophrenia, especially the long form variants [22,25]. Abnormalities in dopaminergic signalling are usually observed in patients with schizophrenia [26]. Porat et al. have suggested that higher concentrations of dopamine can induce apoptosis and increase the expression of TP53 [27]. Therefore, the TP53 gene might be linked to the dopaminergic signalling pathway, even though it is not yet known whether p53 interacts with DRD4. In the study reported herein, our aim was to investigate the role of the TP53 codon 72 polymorphism in schizophrenia. In addition, we investigated the possible interaction between the DRD4 uVNTR and TP53 codon72 polymorphisms.

## Methods

### Participants

For this study, 934 patients with schizophrenia (643 males, average age of 36.69 years, standard deviation (SD) = 12.20) were recruited in Southern Taiwan from outpatient services, the intensive care unit, the community psychiatric clinic, psychiatry referrals, and the acute wards of a teaching hospital. All patients were diagnosed by senior psychiatrists in a teaching hospital according to the criteria of the Diagnostic Statistical Manual, Fourth Edition [28], and were receiving antipsychotic medication when the blood samples were taken. Each patient was assessed independently by at least two psychiatrists on the basis of case records and interviews. The interviews were administered by trained and reliable assessors and the diagnosis depended on the consensus of two assessors. Written informed consent was obtained from all participants and the study protocol was approved by the Institutional Review Board of Kaohsiung Armed Forces General Hospital.

### DNA extraction

DNA was extracted from the blood samples by using a DNeasy Blood and Tissue Kit (Qiagen, Hilden, Germany) according to the instructions of the manufacturer. The extracted DNA was diluted to a final concentration of 100 ng/μl and stored at -80°C until further use.

### Genotyping of the TP53 codon 72 polymorphism

The Pro72Arg polymorphism of TP53 was analysed by the polymerase chain reaction-restriction fragment length polymorphism (PCR-RFLP) method. The PCR was performed using the hot-start technique in a final reaction volume of 25 μl with 1.5 mM MgCl_2_. The primers for PCR amplification were: forward 5'-CAA CGT TCT GGT AAG GAC AA-3'; reverse 5'-GCC TAA GGG TGA AGA GGA A-3'. The following reaction conditions were used: 35 cycles of 30 s at 94°C (denaturation), 30 s at 55°C (annealing), and 30 s at 72°C (extension). The PCR products were digested with *Bst*UI at 60°C for 2 h, and were then visualised on a 2% agarose gel that contained 0.5 μg/ml ethidium bromide. The original undigested PCR product was 488 bp in length. PCR products that corresponded to the Arg72 (CGC) variant were digested into fragments of 222 and 266 bp by *Bst*UI, whereas the Pro72 (CCC)-specific product remained undigested. The genotypes were recorded by at least two well-trained assessors who were blinded to the clinical characteristics of the study sample.

### DNA sequencing

The results of the PCR-RFLP analysis were confirmed by direct sequencing. Briefly, the PCR product was purified using a QIAquick purification column (Qiagen, Valencia, USA) and then used as the template for cycle sequencing with the BigDye Terminator v3.1 Cycle Sequencing Kit (Applied Biosystems, Foster City, USA), according to the instructions of the manufacturer. The extended products were separated on an ABI PRISM™ 3130 Genetic Analyzer (Applied Biosystems, Foster City, USA) after alcohol precipitation.

### Statistical analysis

The variables were analysed using SPSS for Windows version 15.0 (SPSS, Chicago, IL, USA). Continuous variables were shown as means ± SD and categorical variables were presented as proportions. An unpaired *t *test was used to compare continuous variables between groups, and Pearson's chi-square (χ^2^) test was applied to the categorical variables. χ^2 ^tests were used to test whether the genotype frequencies at each locus conformed to Hardy-Weinberg equilibrium. In addition, a χ^2 ^test was used to determine whether intergroup differences in the genotype distributions and allele frequencies of the TP53 codon 72 and DRD4 uVNTR polymorphisms were significant between cases and controls. Moreover, a hierarchical logistic regression analysis with dummy variables was used to evaluate whether the TP53 and DRD4 polymorphisms were independent factors associated with vulnerability to schizophrenia. Possible interaction between the polymorphisms was also analysed in the logistic regression. A total of three dummy variables were created: p53CC, p53GG, and DRD4c. For the variable p53CC, a score of 1 referred to the CC genotype of the TP53 codon 72 polymorphism, whereas 0 referred to the other possible genotypes. For p53GG, a score of 1 referred to the GG genotype of the TP53 codon72 polymorphism and 0 referred to the other possible genotypes. For DRD4c, 1 referred to the long form allelic variants of the DRD4 uVNTR polymorphism (≥ 5 repeats) and 0 referred to the short form allelic variants (≤ 4 repeats). The interrelationships among age and gender of the subjects, genotype CC of the TP53 codon 72 polymorphism, long form variants (≥ 5 repeats) of the DRD4 uVNTR polymorphism, and schizophrenia were analysed by structure equation modelling with AMOS version 7.0 (SPSS). The structural equation modelling techniques used all the information that was provided by the regression techniques and path analysis. If the p value was greater than 0.05 and the adjusted goodness-of-fit index was greater than 0.9, the null model corresponded to the real structure.

## Results

**Figure 1:**
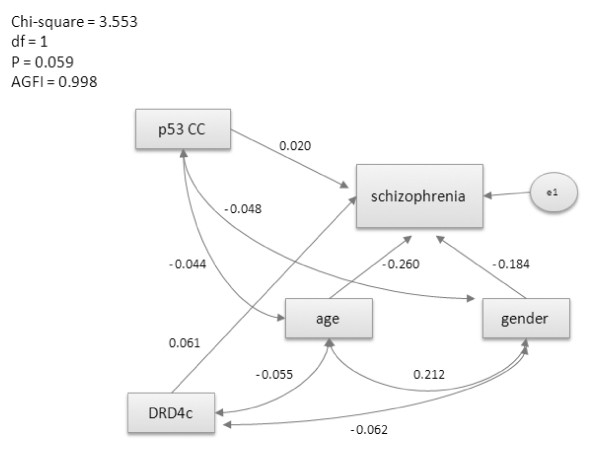
**Structural equation modelling of inter-relationships among variables with respect to schizophrenia**. p53CC: dummy variable where 1 refers to the CC genotype of the TP53 codon 72 polymorphism and 0 refers to the other possible genotypes; DRD4c: dummy variable where 1 refers to the long form allelic variants of the DRD4 uVNTR polymorphism (≥ 5 repeats) and 0 refers to the short form allelic variants (≤ 4 repeats); AGFI: adjusted goodness-of-fit index. The numbers are the coefficients of correlation between two variables.

In this study, the subjects comprised 934 patients with schizophrenia (643 males, average age of 36.69 years, SD = 12.20) and 433 controls from the community (190 males, average age of 45.33 years, SD = 13.91). The distributions of genotypes and alleles for the TP53 codon 72 and DRD4 uVNTR polymorphisms are shown in Table 1. The genotype distribution of the TP53 codon 72 polymorphism conformed to the Hardy-Weinberg equilibrium in both cases and controls (case: HWE-p = 0.884; control: HWE-p = 0.084; Table 1). No significant differences were found for the genotype distribution or allele frequencies of the TP53 codon 72 polymorphism between the cases and the controls (genotype: p = 0.229; allele: p = 0.404). By contrast, the genotype distributions of the DRD4 uVNTR polymorphism did show significant differences between the two groups. In our study cohort, the three most common genotypes of the uVNTR in DRD4 were 4/4, 2/4, and 2/2, and the distribution of these genotypes was significantly different between the cases and the controls (case: 4/4 = 57.00%, 4/2 = 28.77%, 2/2 = 6.56%; control: 4/4 = 66.19%, 4/2 = 18.44%, 2/2 = 11.58%; P < 0.001). All DRD4 uVNTR alleles detected were categorised into the following two groups: short form alleles (≤ 4 repeats) and long form alleles (≥ 5 repeats). In general, alleles that contained more than six repeats were found rarely in our population. Patients with schizophrenia were significantly more likely to carry long form alleles (p = 0.001), and these alleles were associated with a 3.189-fold higher risk of schizophrenia that the short-form alleles (OR = 3.189, 95% CI = 1.535 - 6.622). The results of the logistic regression analysis showed that age and gender were associated with schizophrenia to a significant level (p < 0.001, data not shown); therefore, the subsequent analyses of the genetic effect that was contributed by the two polymorphisms were stratified by age and gender of the subjects. The two dummy variables p53CC and p53GG showed no significant association with schizophrenia after the contributions of age and gender had been taken into account (Model 1 in Table 2; p53CC: p = 0.203; p53GG: p = 0.571). However, the dummy variable DRD4c was associated significantly with schizophrenia (Model 2 in Table 2, p = 0.036, OR = 2.319), which indicated that the long form variants (≥ 5 repeats) confer vulnerability to schizophrenia in our population. In addition, no interaction between the dummy variable DRD4c and p53CC was found (Model 3 in Table 2, p = 0.166). This result might suggest that the long form variants of the uVNTR polymorphism in DRD4 increase the risk of developing schizophrenia in a manner that is independent of the TP53 codon 72 polymorphism. To investigate the possible interaction between the two polymorphisms further, structure equation modelling was used to clarify the inter-relationships between the age and gender of the subject, the dummy variables p53CC and DRD4c, and schizophrenia. The structural model shown in Figure 1 demonstrates that the age and gender of the subjects showed a stronger correlation with schizophrenia than the genetic effect of the two polymorphisms. Age, which showed a negative association with schizophrenia, had the highest correlation coefficient (-0.26), followed by gender (-0.184), DRD4c (0.061), and p53CC (0.020). These results indicated that increasing age and female gender correlate negatively with schizophrenia, which is consistent with the observed higher incidence of schizophrenia among young male individuals. The long form variants (≥ 5 repeats) of the DRD4 uVNTR polymorphism showed a stronger positive correlation with schizophrenia than the CC genotype of the TP53 codon 72 polymorphism. However, both of these genotypes did not parallel to age and gender, which still not reach a significant degree. According to the results of the analysis of pathogenesis, which were consistent with the results of the logistic regression, there was no interaction between p53CC and DRD4c. These findings provide additional evidence that these two polymorphisms are associated independently with schizophrenia. In addition, given that the genetic effect of the TP53 codon 72 polymorphism is very small, it is more likely that this polymorphism is not correlated with schizophrenia.

**Table 1:** The distributions of genotypes and alleles for the TP53 codon 72 and DRD4 uVNTR polymorphisms.

Polymorphism	Genotype frequency (%)											HWE-p	p value	Allele frequency (%)		P value	Odds ratio (95% CI)
**rs8064946**	**C/C**				**C/G**				**G/G**					**C (Pro72)**	**G (Arg72)**		
						
Patient (n = 917)	193 (21.05)				453 (49.40)				271(29.55)			0.884	0.229	839 (45.75)	995 (54.25)	0.404	1.072 (0.910--1.262)^a^
Control (n = 427)	74 (17.33)				228 (53.40)				125 (29.27)			0.084		376 (44.03)	478 (55.97)		
DRD4 uVNTR	2/2	2/3	2/4	2/5	3/3	3/4	3/5	4/4	4/5	4/6	5/6			Short (≤ 4 repeats)	Long (≥ 5 repeats)		
						
Patient (n = 914)	60	0	263	8	1	16	1	521	24	19	1		< 0.001	1774 (97.05)	54 (2.95)	0.001	3.189 1.535--6.622)^b^
Control (n = 423)	49	1	78	3	1	6	0	280	2	3	0			838 (99.05)	8 (0.95)		

Genotyping results for the TP53 codon 72 polymorphism were lacking for 17 patients with schizophrenia and six controls; genotyping information for the DRD4 uVNTR polymorphism was lacking for 20 patients with schizophrenia and 10 controls; HWE-p: p value of chi-square test for Hardy--Weinberg Equilibrium; a: odds ratio of the C allele to the G allele of the codon 72 polymorphism in TP53in relation to schizophrenia; b: odds ratio of the long form variants (≥ 5 repeats) to the short form variants (≤ 4 repeats) shows the former confers enhanced risk of vulnerability to schizophrenia.

**Table 2:** Results of hierarchical logistic regression analysis aimed at determining whether the TP53 codon 72 and DRD4 uVNTR polymorphisms were associated with schizophrenia after the contributions of age and gender were taken into account.

Model 1.						
**Variables**	**B**	**S.E.**	**Wald**	**df**	**Sig.**	**Exp(B)**

Gender	--0.891	0.128	48.735	1	< 0.001**	0.410
Age	--0.045	0.005	83.772	1	< 0.001**	0.956
P53CC	0.218	0.171	1.618	1	0.203	1.244
P53GG	0.083	0.146	0.320	1	0.571	1.086
Constant	3.760	0.276	185.052	1	< 0.001	42.954

**Model 2.**						

**Variables**	**B**	**S.E.**	**Wald**	**df**	**Sig.**	**Exp(B)**

Gender	--0.891	0.128	48.735	1	< 0.001**	0.410
Age	--0.045	0.005	83.772	1	< 0.001**	0.956
DRD4c	0.841	0.401	4.393	1	0.036*	2.319
Constant	3.777	0.270	195.381	1	< 0.001	43.668

**Model 3.**						

**Variables**	**B**	**S.E.**	**Wald**	**df**	**Sig.**	**Exp(B)**

Gender	--0.891	0.128	48.735	1	< 0.001**	0.410
Age	--0.045	0.005	83.772	1	< 0.001**	0.956
DRD4c by p53CC	1.459	1.054	1.916	1	0.166	4.302
Constant	3.819	0.270	200.781	1	< 0.001	45.573

B: coefficient of regression; S.E.: Standard Error; Wald: the index of regression effect; df: degrees of freedom; Sig: P value; Exp(B): odds ratio; p53CC: dummy variable where 1 refers to the CC genotype of the TP53 codon 72 polymorphism and 0 refers to the other possible genotypes; p53GG: dummy variable where 1 refers to the GG genotype of the TP53 codon 72 polymorphism and 0 refers to the other possible genotypes; DRD4c: dummy variable where 1 refers to the long form allelic variants of the DRD4 uVNTR polymorphism (≥ 5 repeats) and 0 refers to the short form allelic variants (≤ 4 repeats); DRD4c by p53CC: the genetic effect attributed to the possible interaction between the long form variants of the DRD4 uVNTR polymorphism and the CC genotype of the TP53 codon 72 polymorphism; ** indicates statistical significance (p < 0.001).

## Discussion

Schizophrenia is a complex trait with a strong genetic background [29,30] whose detailed pathogenesis remains unclear. Recently, several studies have described the epiphenomenon that the incidence of cancer is decreased in patients with schizophrenia compared with that in the general population [31]. In addition, several interesting findings have been proposed to be linked to resistance to tumours in patients with schizophrenia; these include the detection of excess secretion of dopamine, enhanced activity of natural killer cells, and accelerated apoptosis in patients with schizophrenia, together with the unexpected anti-mutagenic effects of antipsychotic drugs [18,31,32]. The p53 gene is also speculated to be involved in resistance to the formation of tumours in individuals with schizophrenia. The induction of apoptosis by p53 and its effects on the cell cycle may account for the neurodevelopmental abnormalities that are associated with schizophrenia, as well as the resistance to tumours. Among the known SNPs in TP53, the CAA Ins/Del and 16 bp Ins/Del polymorphisms were found to be associated with schizophrenia in a case-control study of residents in Toronto. In addition, the CAA Ins/Del polymorphism was shown by transmission disequilibrium test (TDT) analysis to be transmitted unequally in a Portuguese family with schizophrenia [33]. The SNP rs2078486-A allele and a CAC three-marker haplotype CAC were detected more frequently in Chinese patients with schizophrenia, which suggested that TP53 plays a role in susceptibility to schizophrenia [34]. Previously, we have found that the CC (Pro/Pro) genotype of the TP53 codon 72 polymorphism can be detected at a higher frequency in Taiwanese patients with cancer, in particular those with colorectal cancer, than in controls [19,20]. This SNP has been reported to show no association with schizophrenia, the age of onset of schizophrenia [32], or deficits in the neurocognitive profile [35]. However, the role of this polymorphism in Taiwanese patients with schizophrenia remains unclear. In addition, little is known about the possible interactions between the codon 72 polymorphism of TP53 and other SNPs that are related to schizophrenia. The aim of the case-control study described herein was to explore the genetic association between the SNP in codon 72 of TP53, the uVNTR polymorphism in DRD4, and schizophrenia in a Taiwanese population.

We found that the frequency of the Pro72 variant (the C allele) of the TP53 polymorphism was higher in patients with schizophrenia than in controls from the community (45.75% versus 44.03%), which was consistent with the results of a study by Chiu et al. (46.8% vs. 40.5%) [32]. However, these differences were not statistically significant. There was no statistical difference with respect to either genotype or allele frequency between patients and controls after stratification by gender and age. Interestingly, we have shown previously that the Pro72 variant is more prevalent in patients with colorectal cancer (56.1%) or transitional cell carcinoma (62.7%) [19,20]. Taken together, these results suggest that the Pro72 variant is associated with a higher risk of cancer, but not schizophrenia, than the Arg 72 variant. In addition, this finding was consistent with earlier results from two independent case-control studies, which rejected a link between the codon 72 polymorphism in TP53 and increased susceptibility to schizophrenia [18,32]. The question remains as to whether p53 controls neuronal apoptosis as well as serving as a protective factor against cancer in patients with schizophrenia. It is unlikely that a conclusive result will be obtained The answer is likely to be inconclusive, even though the TP53 gene is suspected to be involved in synaptic dysfunction in schizophrenia [36]. Several lines of evidence indicate that the apoptotic activity of p53 might be modulated by polymorphisms in TP53. In particular, the codon 72 polymorphism has been reported to affect the ability of p53 to induce apoptosis. However, although the Pro/Pro genotype has been found to be more frequent in patients with cancer than in controls, it was not significantly less prevalent in patients with schizophrenia than in controls. Hence, our results provide additional evidence that p53 is unlikely to contribute to the resistance to tumours that is observed in patients with schizophrenia.

Schizophrenia is a multi-factorial genetic disease [29,30] that is suggested to be caused by the synergistic effect of different genetic variants [37]. Previously, we have reported that the long form alleles of the uVNTR polymorphism in DRD4 might a risk factor for vulnerability to schizophrenia [25]. Therefore, in the study reported herein, we analysed the possible interaction between the TP53 codon 72 and DRD4 uVNTR polymorphisms. In a cell-based assay, high levels of dopamine can induce apoptosis and increase the level of expression of TP53 [27]. In addition, excess secretion of dopamine and synaptic dysfunction (which is probably the consequence of pro-apoptotic activity in neurons) can be observed in patients with schizophrenia. These findings raise the possibility that dopaminergic signalling regulates both neural differentiation and apoptosis in a dose-dependent manner, and that this activity is related to the function of p53. It has also been suggested that p53 serves as a negative regulator of the dopamine-triggered apoptosis that occurs in response to DNA damage under oxidative stress. However, using different statistical models, we did not find any interaction between these two polymorphisms that was associated with schizophrenia. Our results suggested that long form variants of the DRD4 uVNTR polymorphism and genotype CC of the TP53 codon72 polymorphism might not have a synergistic effect on the risk of schizophrenia. In addition, given that the genetic effect of the CC genotype of the TP53 codon 72 polymorphism on schizophrenia is small, this genotype is unlikely to be associated with schizophrenia.

Our results should be viewed in the light of certain limitations. Firstly, with respect to the SNPs in TP53, we only explored the association between the codon 72 polymorphism of TP53 and schizophrenia, as well as its possible interaction with long form variants of the uVNTR polymorphism of DRD4. We did not analyse other SNPs in TP53 that have been reported elsewhere to be associated with schizophrenia [33,34]. A relative few markers across the p53 gene plausibly do not fully determine its role in schizophrenia, of particular, its function involves in apoptosis induction. Secondly, this was a case-control study, rather than a family-based study, and the data obtained might be insufficient to clarify the association between the gene and the disease. For example, we were not able to obtain information about the rate of transmission or linkage disequilibrium. Thirdly, given that no non-Asian subjects were recruited for this study, ethnic heterogeneity can only be identified through a meta-analysis. However, we did not perform a meta-analysis to distinguish whether the results obtained might be false negative findings due to population stratification. Finally, schizophrenia is a complex disorder and it needs to be studied by multifaceted approaches, not solely by the analysis of SNPs.

## Conclusions

The VNTR polymorphism in DRD4 has been reported previously to be involved in the pathogenesis of schizophrenia [19,22]. The Pro72Arg polymorphism in TP53 has been associated with the metastasis of tumours in colorectal cancer and transitional cell carcinoma [19,20], but not with schizophrenia. In addition, the polymorphism is associated with the grade of malignancy rather than onset of the disease. In the present study, we found that the uVNTR polymorphism in DRD4 was associated with vulnerability to schizophrenia, but the Pro72Arg polymorphism in TP53 was not. No interaction between the two polymorphisms was found in the statistical model. Therefore it is necessary to investigate whether other SNPs in the TP53 gene or SNPs in other apoptosis-related genes play a role in the synaptic dysfunction that is involved in the pathogenesis of schizophrenia.

## Competing interests

The authors declare that they have no competing interests.

## Authors' contributions

All authors contributed to the design of the study. FW conceived the study, performed the statistical analysis, and helped to draft the manuscript. BC helped to draft the manuscript. WT carried out the molecular genetic studies and drafted the manuscript. CN, YC, and DS collected the data, coordinated the study, and helped to draft the manuscript. All authors have revised the manuscript and have approved the final manuscript.

## Pre-publication history

The pre-publication history for this paper can be accessed here:

http://www.biomedcentral.com/1471-2350/10/147/prepub
